# A map of African humid tropical forest aboveground biomass derived from management inventories

**DOI:** 10.1038/s41597-020-0561-0

**Published:** 2020-07-08

**Authors:** Pierre Ploton, Frédéric Mortier, Nicolas Barbier, Guillaume Cornu, Maxime Réjou-Méchain, Vivien Rossi, Alfonso Alonso, Jean-François Bastin, Nicolas Bayol, Fabrice Bénédet, Pulchérie Bissiengou, Georges Chuyong, Benoît Demarquez, Jean-Louis Doucet, Vincent Droissart, Narcisse Guy Kamdem, David Kenfack, Hervé Memiaghe, Libalah Moses, Bonaventure Sonké, Nicolas Texier, Duncan Thomas, Donatien Zebaze, Raphaël Pélissier, Sylvie Gourlet-Fleury

**Affiliations:** 1 AMAP, Univ Montpellier, IRD, CNRS, INRAE, CIRAD, Montpellier, France; 2 CIRAD, UPR Forêts et Sociétés, F-34398 Montpellier, France; Université de Montpellier, F-34000 Montpellier, France; 3 CIRAD, UPR Forêts et Sociétés, Yaoundé, Cameroun; 4 Center for Conservation and Sustainability, Smithsonian Conservation Biology Institute, National Zoological Park, Washington, DC USA; 5 CAVELab Computational and Applied Vegetation Ecology, Department of Applied Ecology and Environmental Biology, Faculty of Bioscience Engineering, Ghent University, Ghent, 9000 Belgium; 6 FRM Ingénierie, 34130 Mauguio – Grand Montpellier, France; 7 Institut de pharmacopée et de médecine traditionnelle (Herbier National du Gabon), CENAREST, Libreville, Gabon; 8 Department of Plant Science, Faculty of Science, University of Buea, Buea, Cameroon; 9 Terre Environnement Aménagement, 13001 Marseille, France; 10 Forest is Life, TERRA Teaching and Research Centre, Gembloux Agro-Bio Tech, University of Liège, Gembloux, Belgium; 11 Plant Systematic and Ecology Laboratory (LaBosystE), Department of Biology, Higher Teachers’ Training College, University of Yaoundé I, P.O. Box 047, Yaoundé, Cameroun; 12 ForestGEO, Smithsonian Tropical Research Institute, NMNH - MRC 166, P.O. Box 37012, Washington, DC 20013-7012 USA; 13 Institut de Recherche en Ecologie Tropicale/Centre National de la Recherche Scientifique et Technologique, Libreville, Gabon; 14 Faculty of Sciences, Evolutionary Biology and Ecology, Université Libre de Bruxelles, CP160/12, 50 Av. F. Roosevelt, 1050 Brussels, Belgium; 15 School of Biological Sciences, Washington State University, 14204 NE Salmon Creek Ave, Vancouver, WA 98686 USA

**Keywords:** Environmental sciences, Forestry, Ecology, Climate sciences

## Abstract

Forest biomass is key in Earth carbon cycle and climate system, and thus under intense scrutiny in the context of international climate change mitigation initiatives (e.g. REDD+). In tropical forests, the spatial distribution of aboveground biomass (AGB) remains, however, highly uncertain. There is increasing recognition that progress is strongly limited by the lack of field observations over large and remote areas. Here, we introduce the Congo basin Forests AGB (CoFor-AGB) dataset that contains AGB estimations and associated uncertainty for 59,857 1-km pixels aggregated from nearly 100,000 ha of *in situ* forest management inventories for the 2000 – early 2010s period in five central African countries. A comprehensive error propagation scheme suggests that the uncertainty on AGB estimations derived from c. 0.5-ha inventory plots (8.6–15.0%) is only moderately higher than the error obtained from scientific sampling plots (8.3%). CoFor-AGB provides the first large scale view of forest AGB spatial variation from field data in central Africa, the second largest continuous tropical forest domain of the world.

## Abstract (summary)

**Table:** 

Measurement(s)	organic material • aboveground biomass
Technology Type(s)	digital curation
Sample Characteristic - Environment	forested area
Sample Characteristic - Location	Central Africa

Machine-accessible metadata file describing the reported data: 10.6084/m9.figshare.12504911

## Background & Summary

Quantifying forest aboveground biomass (AGB) has been the focus of considerable interest in the last century for both commercial (e.g. timber, fiber, energy productions) and scientific (e.g. forest ecosystem functioning and productivity)^1^ purposes. Nowadays, forest AGB is at the forefront of international research and political agendas due to its importance in Earth climate system and carbon cycle^2,3^. While most of global forest AGB is found in tropical regions^4^, its spatial distribution within the main tropical forest basins remains subject to large uncertainties, even at regional scale^5^. This knowledge gap introduces uncertainties into estimates of forest carbon release from land-use change^6,7^, and constitutes a major impediment to the design and implementation of climate change mitigation policies (e.g. Reducing Emissions from Deforestation and forest Degradation, REDD) into countries national development planning. Given the escalation of carbon emissions associated with deforestation^8^ and forest degradation^9^, fires^10^ and drought events^11^ in the tropics, it is more than ever important to increase our understanding of tropical forest AGB geography and of its ecological determinants.

Historically, forest resources assessments have been made through ground inventories^12^. In temperate countries where forest management planning is a long-standing tradition, national forest inventories (NFI) have been established early-on and are regularly updated. Across western Europe for instance, today’s sampling intensity is such that it allows visualizing spatial variations of forest AGB from plots AGB estimations alone^13^, and thus inferring reliable mean AGB levels at various jurisdictional scales^14^. In most tropical countries, however, national forest resources assessment is a more recent practice facing multiple challenges, including limited financial resources, difficulties to access remote regions, and the lack of initial information on forest resource distribution to rationalize any new sampling plan. In the Democratic Republic of Congo for example, which extends on c. 2.345 M.km^2^ and hosts the majority of the Congo Basin forests, the first NFI was initiated in 2017 and aims at establishing about 400 sampling units^15^, while a country like Germany set up more than 40,000 sampling units^16^ for an area of c. 0.357 M.km^2^. While DRC’s NFI is a tremendous undertaking and an important step forward for this country, it is obvious that it will only provide a crude picture of forest AGB spatial distribution. To address the limits of inventory-based estimation methods in such context, a frequent strategy consists of using field sample plots established by research teams (hereafter referred to as scientific plots) to build and validate AGB prediction models based on remote sensing and environmental data^17,18^. Yet again, the representativeness of field plot estimates generally is a major limit that potentially introduces significant biases in model outputs^5^. Indeed, large remote areas are usually unsampled in the tropics so that broad environmental gradients, which may both have an impact on forest AGB and on the remote sensing signal, are not fully represented in these datasets.

Despite pressing needs for field data on tropical forest AGB stocks, management forest inventories have remained overlooked by the scientific and greenhouse gas inventory communities. Since the United Nations summit in Rio de Janeiro (1992), central African countries have begun to revise their forest laws to achieve sustainable forest management, notably making forest management planning mandatory in production forests^19^. In 2019, 58% of about 50 M.ha of timber logging concessions have been inventoried for management purposes^20^, with sampling rates of about 1% depending on countries’ forest laws. These data thus contain information on forest structure and composition over areas that are several orders of magnitude larger than the current extent of scientific forest inventories in the region, which hosts the second largest block of continuous tropical forest on Earth.

**Fig. 1:**
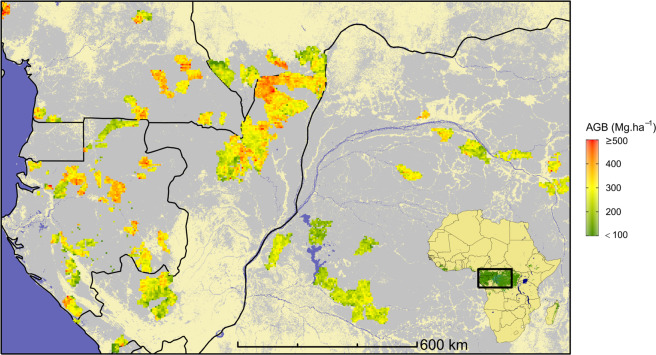
Overview of the study area in central Africa and field data distribution. Reference forest AGB estimates derived from field inventories, aggregated into 5-km pixels (for visual purpose), are depicted in a green-to-red color gradient and underlaid by the spatial distribution of moist forests^21^ (grey) and countries borders (black).

**Fig. 2:**
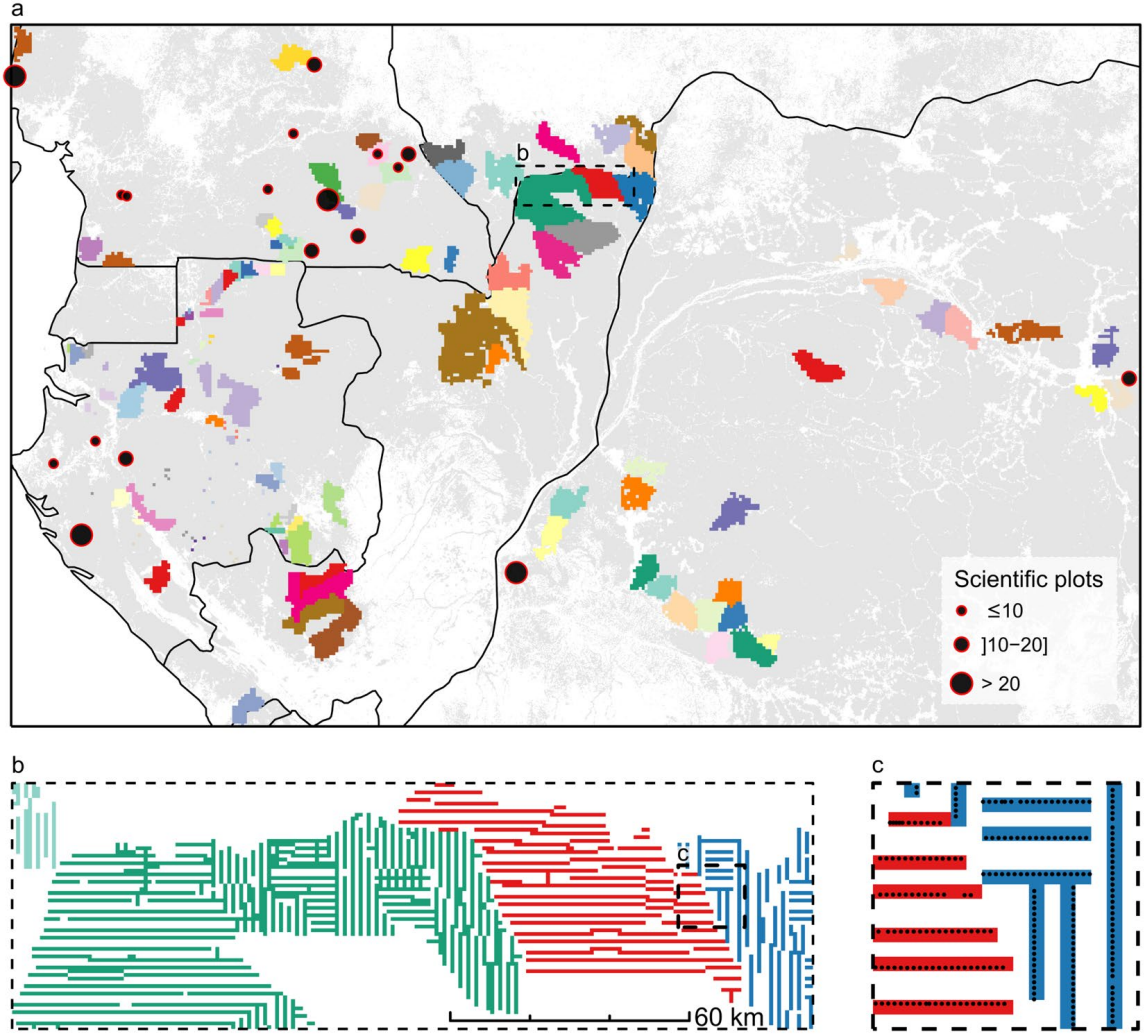
Spatial distribution of forest inventory data in managed concessions of central Africa. (**a**) Inventory data are colored by logging concessions and aggregated into 5-km pixels (for visual purpose). Black dots represent the location of scientific plots used to evaluate the biomass computation scheme, with dots’ size corresponding to the number of plots per sampling site. Forestry data is underlaid by the spatial distribution of moist forests^21^ (light grey) and national limits (black). Outlined region is expanded in **b**. (**b**) Selected subset of forest management inventory data colored by logging concessions and displayed at the actual scale of CoFor-AGB, i.e. 1 km. Outlined region is expanded in **c**. (**c**) Forest management inventory plots (black dots) along inventory transects, underlaid by the 1-km pixel grid used to aggregate inventory data and compute forest biomass.

In this paper, we give access, thanks to the voluntary participation of several logging companies, to nearly 60 thousand 1-km resolution AGB estimates and associated uncertainty spread over contrasted forest types and environmental conditions in five countries from central Africa (Fig. 1). These estimates are based on the aggregation of c. 11.8 million measured trees in c. 192,000 field plots, representing an estimated inventory workload of 1000 years for a single person. We validated the AGB computation scheme using data from scientific plots (Fig. 2), allowing a detailed characterization of uncertainty sources and their propagation to plot-level estimations. This dataset thus provides the largest ever published dataset on field-derived tropical forest AGB giving unprecedented information on both the local and regional variability of AGB in tropical forests.

## Methods

### Data presentation

#### Managed concessions forest inventory data

The forest inventory dataset (hereafter CoFor dataset) corresponds to forest management inventories from 114 logging concessions (Fig. 2). Inventories followed standardized protocols^22^ and were conducted from the early 2000s to the early 2010s, at the exception of the Ngotto concession (1.4% of the data, sampled in 1993–1996) in the Central African Republic. Sampling designs were systematic and usually consisted of 20 to 25 m wide continuous and parallel transects, located 2 to 3 km apart, that were then aggregated into plots of 0.4 ha (8% of the cases) or 0.5 ha (91.7% of the cases). The remaining 0.3% of the plots ranged between 0.3 and 1 ha. Within each plot, information on tree diameter at breast height (DBH) and taxonomic identity were available for all trees with DBH ≥ 30 cm while smaller trees were either partially sampled or in some cases completely missing (see further). The quality of taxonomic information has been extensively reviewed elsewhere^22^. In total, the CoFor dataset contained 191,562 plots, covering a cumulated sampling area of 94,513 ha and representing 11.82 × 10^6^ tree measurements and 1,091 identified taxa.

#### Scientific forest inventory data

We used scientific forest inventory data to fine-tune and test the AGB computation scheme applied to forest management inventories and described in the next section. Scientific forest data were collated from plot networks and collaborators in Cameroon (IRD plot network^23^, CTFS Korup^24^ plot), Gabon (CTFS Rabi^24^ plot) and D.R. Congo^25^ (Fig. 2), and represented 233 1-ha plots. In each plot, the DBH of all trees with DBH ≥ 10 cm was measured and trees were identified following standard scientific protocols^26^. Forest structure in the scientific dataset covered most of the structural range found in CoFor, at the exception of forests characterized by both a small mean tree size and a low tree abundance (Supplementary Fig. 1), likely reflecting degraded forest states which can notably be found in Northern Congo (the so-called Marantaceae forests).

### Estimation of plot and pixel aboveground biomass

#### Caveats of management inventories in forest concessions

Although the amount of information on central African forests structure and composition in the CoFor dataset is of unprecedented size and spatial representativeness, data collection protocols followed by forest companies do not conform to standard scientific protocols. This entailed additional computation steps and uncertainty sources when estimating forest sample plots aboveground biomass (AGB).

**Fig. 3:**
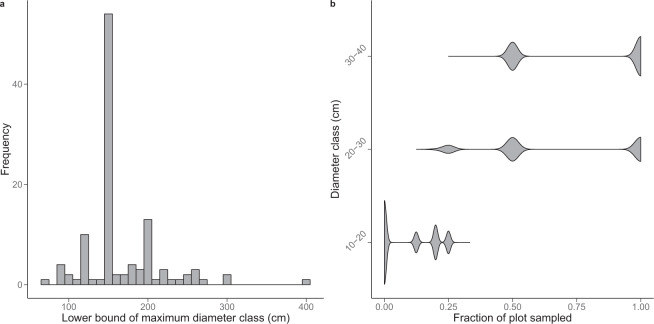
Caveats of forest management inventory data. (**a**) Frequency distribution of maximum diameter classes found across the 114 commercial inventories. For a given inventory, a maximum diameter class of e.g. 150 cm indicates that either 150–160 cm is the largest tree diameter class found in the field, or 150 cm is the opening threshold of the right-censored diameter distribution. (**b**) Frequency distributions of sampling rates (expressed as a plot fraction) for the three firsts diameter classes. For about half of the plots, trees in diameter class 10–20 cm were not sampled (i.e. plot fraction of 0), while trees in diameter class 30–40 cm were sampled on the entire plot area (i.e. plot fraction of 1). From diameter class 40–50 cm onward, trees were always sampled on the entire plot area.

The first limitation of forest management inventories is that tree diameters are recorded as discrete (i.e. by 10 cm width bins), censored data rather than continuously. Tree diameter distributions were right-censored, meaning that all trees above a certain diameter threshold were pooled in a single ‘open class’. This diameter threshold varied across the 114 logging concessions (Fig. 3a) and mainly corresponded to 120 cm, 150 cm or 200 cm (8.9%, 47.8% and 11.5% of the sampling plots, respectively). Since tree AGB allometric models require continuous tree diameter values, it was necessary to establish a strategy to derive continuous DBH values from diameter classes (hereafter *specific function 1*).

Secondly, while a standard practice in scientific inventory protocols is to census all trees with DBH ≥ 10 cm, this census threshold can be higher in forest management inventory protocols and trees in the smallest diameter classes are not always sampled on the entire plot. In the CoFor dataset, for instance, the tree census threshold was 20 cm DBH in about half of the plots (i.e. 51.6%) and 10 cm DBH otherwise. Trees in the 20–30 and 30–40 cm DBH classes were sampled on the entire plot area in about half of the sampling plots (i.e. 42.7% and 58.4%, respectively) and partially sampled, most often on half of the plot area (Fig. 3b), in the remaining cases. When present, information on trees in the 10–20 cm DBH class was the least complete, as it typically corresponded to a sampling of 12.5% up to 33% of the plot area (Fig. 3b). Since forest AGB is commonly reported for all trees ≥ 10 cm DBH in the scientific literature, we developed procedures to correct for the partial inventory (hereafter *specific function 2*) or complete absence (hereafter *specific function 3*) of small trees in forest management inventories.

#### Computation scheme

**Fig. 4:**
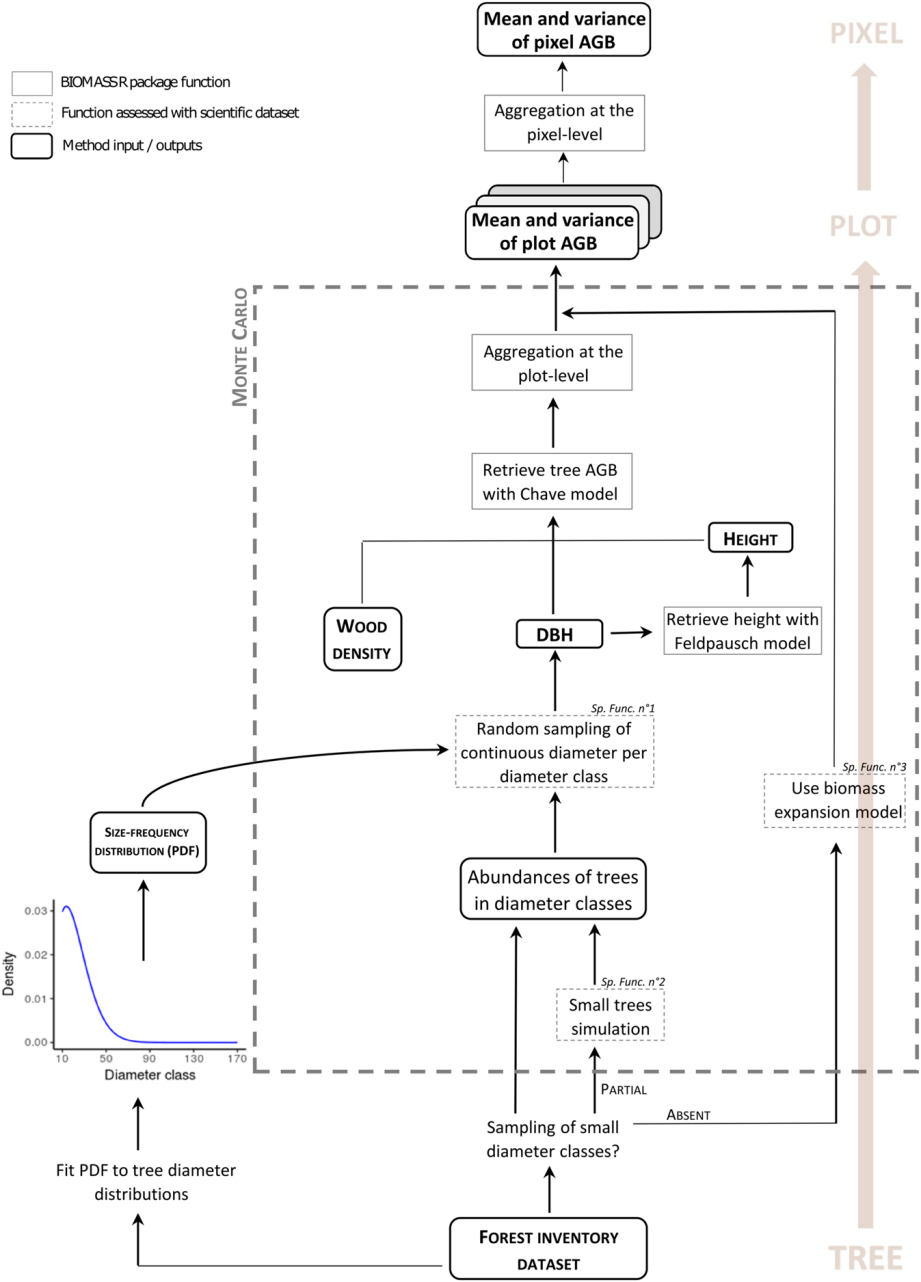
Workflow of aboveground biomass (AGB) computation scheme from forest management inventory data to plot and pixel estimations. The three *specific functions* (noted *Sp. Func*.) developed for this study are framed with dashed lines (see sections below for details).

We built a complete computation scheme to derive standardized estimates of plot AGB and associated errors from the CoFor dataset using a Monte Carlo approach (see Fig. 4 for a general overview). The general workflow can be decomposed into three main steps. First, forest management inventory data were transformed so as to mimic the format of scientific inventory data, which implied to (i) simulate the abundance of small trees in partially-sampled diameter classes (*specific function 2*) and (ii) assign continuous tree diameters from discrete diameter classes (*specific function 1*). Second, we used the BIOMASS R package^27^ for computing standardized plot AGB from inventory data using the pantropical AGB allometric model^28^ and for error propagation. Package’s build-in functions were used to retrieve tree wood density from the Global Wood density database^29^ and tree height using Feldpausch’s height-diameter allometric model^30^ parametrized for the central African region. An estimate of plots AGB was then obtained from trees AGB and either corresponded to the AGB above 10 cm DBH (when trees in the 10–20 cm DBH class were partially sampled in the plot) or above 20 cm DBH (when trees in the 10–20 cm DBH class were not sampled in the plot). In this last case, a third step consisted in applying a correction to plots AGB estimations (i.e. *specific function 3*) to predict plots AGB above the standard 10 cm DBH threshold.

For each plot, we generated 1000 AGB estimates using a Monte Carlo procedure. For each Monte Carlo simulation, errors associated to each computational step are calculated and propagated throughout the computation chain. This approach has been described elsewhere, notably in the BIOMASS R package^27^, and is now used as a standard for generating calibration AGB data for satellite missions^31^. The procedure outputs a vector of AGB estimates for each plot (of length 1000 here) from which we extracted the mean and the variance. The mean of AGB estimates within plots was used as the plot AGB value, while the variance and the coefficient of variation (CV) were used to compute estimation uncertainty. Besides classical error sources handled by the BIOMASS R package, an important aspect of our preliminary analyses was to quantify the errors associated to the three *specific functions* introduced below to deal with the peculiarities of forest management inventory data and for which we used scientific forest inventory as test data (see Technical Validation section).

Once computed, plot-level AGB estimates were aggregated into 1-km pixels using the weighted mean function of the SDMTools R package^32^, with plots area as the set of weights. The resulting 59,857 pixels map represented in Fig. 1 provides the first overview of large-scale spatial variation in African moist forests AGB derived from field data. Anticipating users’ needs for pixel-level AGB uncertainty assessments, we also computed (i) the weighted mean of within-pixel plots AGB variance (corresponding to intra-plot variance) and (ii) the weighted variance of within-pixel plots mean AGB (corresponding to inter-plot variance). Summing the intra- and inter-plot variances yields pixel-level total variance, which we expressed as a coefficient of variation (in %) to report on the total AGB uncertainty at the pixel scale. Intra- and inter-plot variances were also separately expressed as coefficients of variation (in %), and are hereafter referred to as estimation uncertainty and sampling uncertainty, respectively.

#### Specific function 1: assignment of tree diameters from diameter classes

Assigning continuous tree diameter values from a discrete, censored distribution requires positing (i) a probability distribution function (PDF) for continuous tree diameters within each diameter class and (ii) the maximum diameter (D_max_) a tree may reach in the open diameter class (e.g. 150–D_max_ cm). In preliminary testing, we used the forest management inventory dataset to fit three probability distribution functions, namely an exponential distribution, a two-parameter Weibull distribution and a two-parameter Gamma distribution, each time considering a set of realistic D_max_ values (i.e. from 300 to 500 cm by 50 cm steps). Distribution functions were fitted either by genus or considering all genera simultaneously. A weighted AIC was used to select the distribution that best fitted the data. We then assessed how well these different strategies (i.e. genus-specific or generic PDFs, distribution function type) allowed estimating actual plot AGB using the set of scientific forest plots. Continuous tree diameters in the scientific inventory dataset were discretized, and we used the fitted PDFs to generate continuous tree diameter by inverse transform sampling^33^. We then compared plot AGB estimates derived from degraded and original data. Based on 1000 iterations of the Monte Carlo scheme on the scientific dataset, we found that D_max_ value had little influence on the results and that using genus-specific PDFs rather than a single generic PDF did not significantly reduce AGB estimation errors. In the final AGB computation scheme (Fig. 4), we thus arbitrarily set D_max_ to 400 cm and selected the best generic PDF, i.e. the 2-parameter Weibull distribution function, with a scale parameter λ = 8.593 and a shape parameter k = 0.737.

#### Specific function 2: simulation of small tree data in partially-sampled diameter classes

Forest management inventory data often contain a partial sampling of trees in the smallest diameter classes, i.e. trees in diameter class *i* are sampled in a spatial fraction *p* of the plots, with *i* and *p* varying across logging companies (Fig. 3b). To compute plot AGB for all trees ≥ 10 cm DBH, it was necessary to account for trees that were not sampled. Here, we developed a two-step procedure to simulate missing tree data: at each iteration of the plot AGB Monte Carlo computation, we (i) expanded the tree count *r* observed in a given diameter class and plot from the spatial fraction *p* to the entire plot by randomly generating a value from a negative binomial distribution X ~ NegBin(*r*, *p*), and (ii) assigned species labels to simulated trees at the pro-rata of species abundance observed in the *p* fraction of the plot.

#### Specific function 3: biomass correction model for trees in diameter class 10–20 cm

**Fig. 5:**
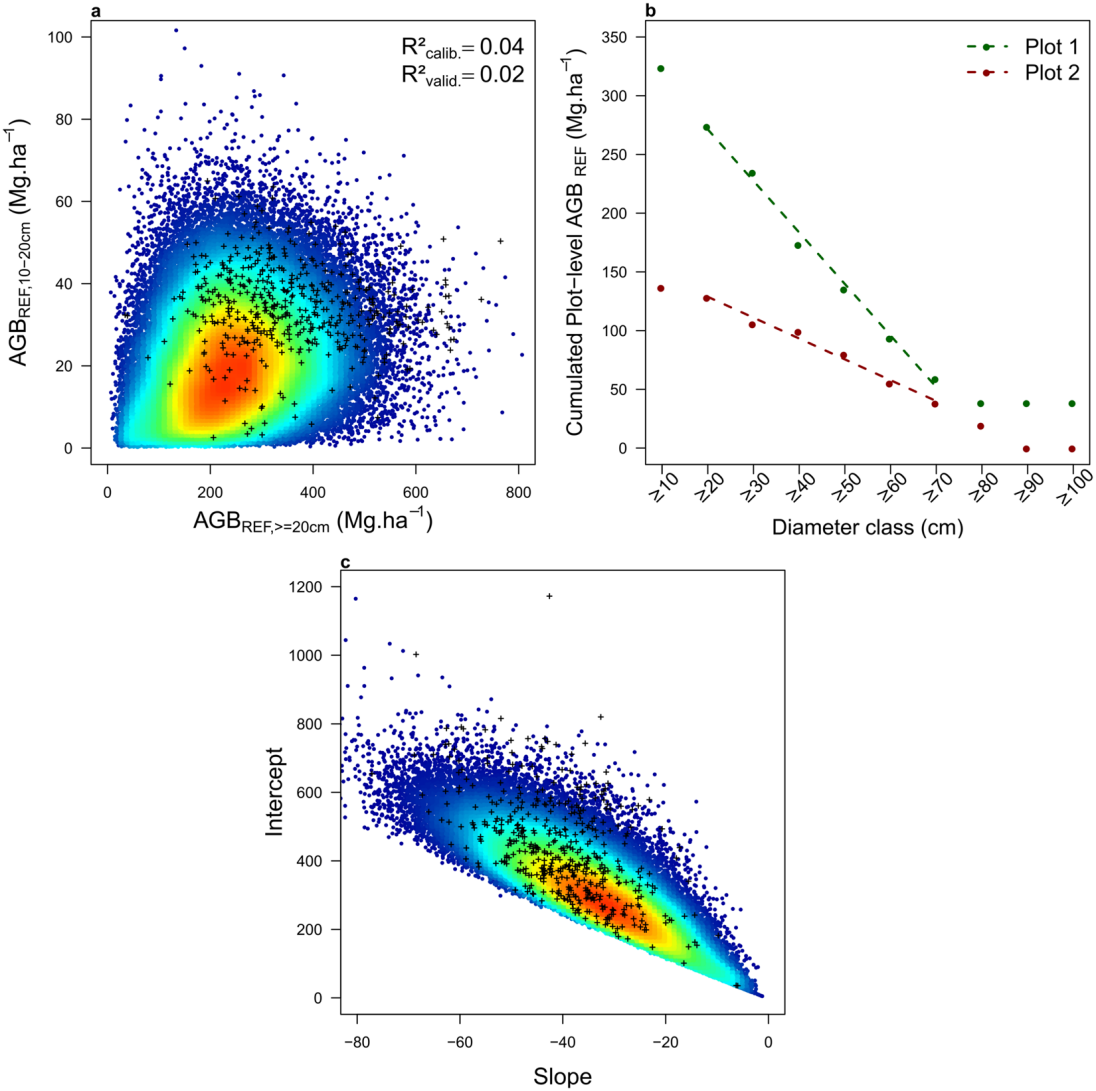
Biomass correction model for trees in diameter class 10–20 cm. (**a**) Heat plot showing the relationship between tree AGB in the 10–20 cm diameter class *vs* AGB of trees ≥ 20 cm diameter in commercial inventories, overlaid by the scientific dataset (small black crosses). (**b**) Shape of the cumulated AGB distribution across 10 cm wide diameter bins for two illustrative plots, with dashed lines representing the fit of the linear models of slope *S* and intercept *I*. (**c**) Heat plot showing the relationship between *S* and *I* in commercial inventories, overlaid by the scientific dataset (small black crosses).

To predict the total biomass of plots where trees in diameter class 10–20 cm were not sampled, we developed a biomass correction model using c. 90,000 forest management plots where trees in the 10–20 cm diameter class were partially sampled. In preliminary testing, we first computed AGB of trees in the 10–20 cm diameter class (AGB_10–20_) and above 20 cm (AGB_≥20_) for each plot, as the average of 1000 AGB simulations while propagating classical errors (on wood density, height-diameter and AGB allometric models) as well as errors on the simulation of small trees and diameter assignment functions inherent to our AGB Monte Carlo computation scheme. Across forest management plots, AGB_10-20_ represented on average 10.1 ± 8.6% of AGB_≥20_, close to what was found on the scientific dataset (11.3 ± 7.9%). Then, we tested several simple linear models to predict AGB_10-20_, using the set of 90,000 plots as calibration data and scientific plots as validation data. Using AGB_≥20_ as sole model predictor led to a poor calibration fit (R²_calib._ of 0.04, Fig. S5a) and predictive power (R²_valid._ of 0.02 on scientific plots). In order to improve the AGB_10-20_ model, we thus computed two additional metrics, *S* and *I*, describing the shape of plots cumulated biomass distribution function (Fig. 5b). *S* and *I* correspond respectively to the slope and the intercept of a simple linear model calibrated on plots’ cumulated biomass by 10 cm classes, from 20 cm up to 70 cm. The distribution of *S* and *I* on commercial data was consistent with that found on scientific data (Fig. 5c). We found that including *S* and *I* in the AGB_10-20_ model led to a significant improvement (although modest) of its calibration fit (R²_calib._ of 0.18) and predictive power (R^2^_valid_. of 0.29 on scientific plots). In the final AGB computation scheme (Fig. 4), we therefore retained this model to predict AGB_10-20_:

$$AG{B}_{10-20}=a+b\ast AG{B}_{\ge 20}+c\ast S+d\ast I+\varepsilon $$

where *a*, *b*, *c* and *d* are model coefficients and *ε* is the error term, assumed to follow a normal distribution N ~ (0, *ε*²), with *ε* the residual standard error of the model.

## Data Records

The CoFor-AGB dataset is distributed as a five layers GeoTIFF file through figshare^34^. The file projection system is World Mercator (EPSG: 3395).

Layer 1: Mean pixel AGB (in Mg.ha^−1^)

Layer 2: Number of field plots within the pixel

Layer 3: Mean date of plots inventory, expressed in fractional year

Layer 4: Inter-plot variance (in Mg^2^.ha^−2^)

Layer 5: Intra-plot variance (in Mg^2^.ha^−2^)

Note that additional information on inventory data and sources are available at plot-level for user’s convenience^35^. To access tree-level inventory data, researchers must send a request to the CoFor consortium (CAFInv@cirad.fr) specifying (*i*) the identification number(s) of the plot(s) they wish to access to, (*ii*) a research project description, detailing the research objectives and planned analyses and (*iii*) a description of the data required. If the request is approved, researchers’ host Institution will be asked to sign a Data Usage Agreement form.

## Technical Validation

### Validation of the plot aboveground biomass computation scheme

Since uncertainties propagation from trees to plot depends on the number of trees (hence plot size), 1-ha scientific plots were split in two approximately equal parts so as to mimic the dominant size of commercial inventory plots (i.e. 0.5-ha), resulting in 466 plots of 0.48-, 0.50- or 0.52-ha depending on the spatial information available in scientific inventories (i.e. quadrat size). Scientific plot data were then degraded to match the level of information found in commercial plot data (e.g. discretization of continuous DBH distributions) and plot AGB estimates obtained from degraded and original datasets were compared to quantify systematic and random error components associated to focal functions (e.g. the assignation of continuous tree diameters from discrete diameter bins) or to several functions combined (e.g. assignation of continuous diameters and simulation of small trees abundance).

**Fig. 6:**
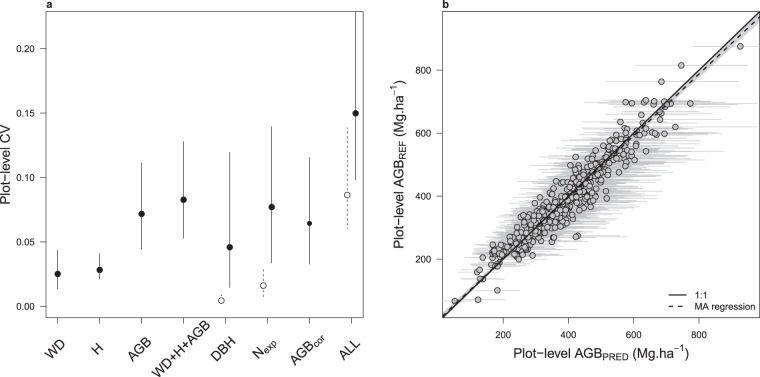
Validation of the AGB computation scheme with scientific inventory data. (**a**) Mean ± 95% confidence interval of plot-level coefficient of variation (CV) on AGB estimation when propagating a single (WD: wood density, H: height-diameter model, AGB: biomass model, DBH: continuous tree diameter assignment function, N_exp_: small tree simulation function, AGB_cor_: biomass correction model for trees in 10–20 cm DBH class) or several error sources combined. For the assignment of tree DBH, we simulated two contrasted scenarios corresponding to a diameter threshold for the open diameter class of 200 cm (open circles) and 120 cm (full circles). For the simulation of small trees, we simulated the two most contrasted scenarios found in commercial data, namely (i) a partial inventory on diameter class 10-20 cm only (over 25% of plot area, open circles) *vs* (ii) a partial inventory on diameter classes 10–20, 20–30 and 30–40 cm (over 12.5, 12.5 and 25% of plot area, respectively, full circles). We finally propagated all error sources (noted ALL) following the best (open circles) and worst (full circles) case scenarios. In the latter case, trees in the 10-20 cm diameter class were completely removed and the AGB correction model for this class was used. (**b**) Plot-level AGB estimates of scientific reference data against predicted AGB based on degraded data so as to match the worst-case scenario (noted ALL in the left panel) found in commercial inventories. Grey dots and bars represent the mean and 95% confidence interval of plots biomass, respectively.

Based on 1000 iterations of the Monte Carlo procedure, we found that computing plot AGB from raw scientific data led to an uncertainty of 8.3% on average (95% CI: 5.2–13.1) when propagating classical uncertainty sources related to wood density, height-diameter and AGB allometric models. When considering as well the errors associated to (i) the assignment of continuous tree diameters from discrete diameter classes (i.e. *specific function 1*), (ii) the simulation of small trees in partially-sampled diameter classes (i.e. *specific function 2*) and (iii) the correction of plot AGB when trees in the 10-20 cm DBH class were not sampled (i.e. *specific function 3*), the average uncertainty on plots AGB ranged from 8.6% (6.0–13.9) in the ‘best-case scenario’ to 15.0% (9.8–23.3) in the ‘worst-case scenario’ (see Fig. 6a). The best-case scenario simulates a situation where a relatively high level of detail is available in forest management inventory data: a large diameter threshold for the open class of the diameter distribution (i.e. 200 cm), a full sampling of trees in diameter classes 20–30 and 30–40 cm and a sampling of trees in diameter class 10–20 cm over 25% of the plot area. The worst-case scenario simulates the opposite situation: a low diameter threshold for the open class of the diameter distribution (i.e. 120 cm), a partial sampling of trees in diameter classes 20–30 cm (over 12.5% of the plot area) and 30–40 cm (over 25% of plot area) and no sampling of trees in diameter class 10–20 cm. Even in the worst-case scenario, the relationship between plot AGB estimates and reference AGB was unbiased (slope of the Major Axis (MA) regression: 0.98 [0.95–1.01], Fig. 6b).

### Validation of the tree diameter assignment function (specific function 1)

**Fig. 7:**
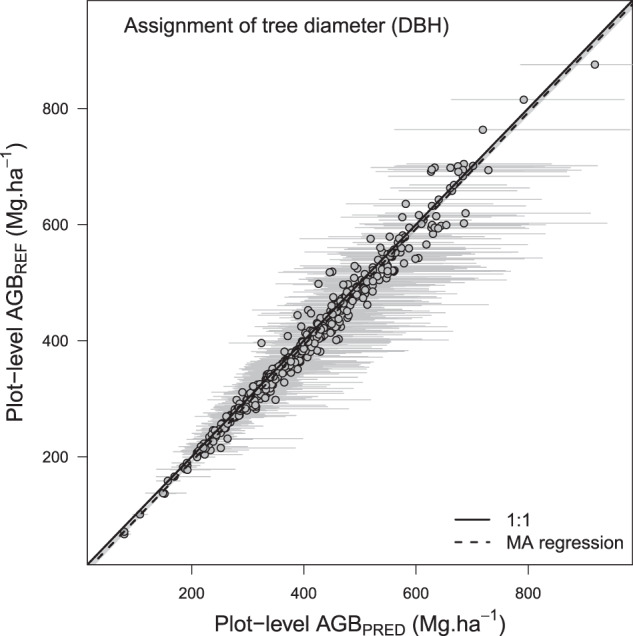
Results of the tree diameter assignment function. AGB_REF_ corresponds to an average of 1000 plots AGB simulations while propagating wood density, height-diameter and allometric model errors. In AGB_PRED_ (mean ± SD), the error associated with the tree diameter assignment function is also propagated. Grey dots and bars represent the mean and 95% confidence interval of plots biomass, respectively.

Using the generic exponential PDF, the assignment of continuous diameters from discrete diameter classes propagated a relatively moderate uncertainty to plot-level AGB that varied with the position of the open diameter class. For instance, the average uncertainty was 0.4% (0.2–0.8) and 4.6% (1.4–12.0) when tree diameters above 200 cm and 120 cm were pooled in a single ‘open class’ (Fig. 6a), respectively. The tree diameter assignment function did not propagate a systematic error across the range of plots’ AGB (slope of the MA regression: 1.00 [0.98–1.01], Fig. 7)

### Validation of the small tree simulation function (specific function 2)

**Fig. 8:**
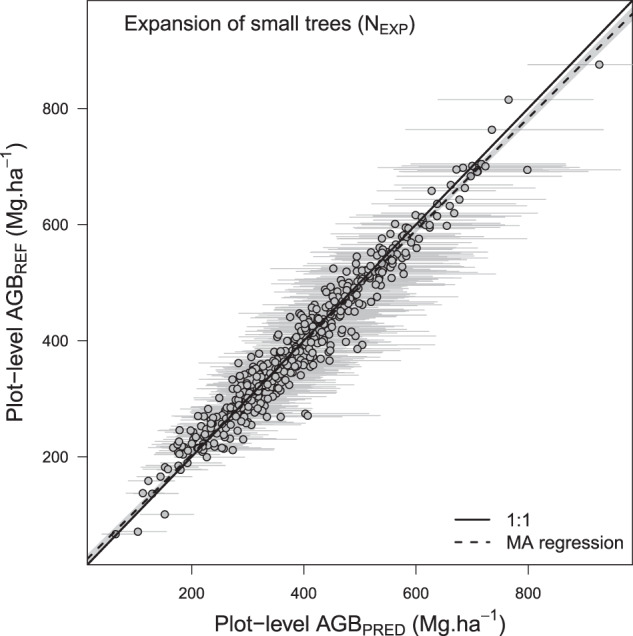
Results of the small tree simulation function. AGB_REF_ corresponds to an average of 1000 plots AGB simulations while propagating wood density, height-diameter and allometric model errors. In AGB_PRED_ (mean ± SD), the error associated with the small tree simulation function is also propagated. Grey dots and bars represent the mean and 95% confidence interval of plots biomass, respectively.

The small tree simulation procedure was evaluated using the scientific dataset by removing information on small trees so as to match varying sampling scenarios observed in commercial data. Figure 8 shows the results of the procedure (AGB_PRED_ based on expanded small tree abundance, N_EXP_) against reference AGB (i.e. computed on full scientific data) when simulating the lightest sampling of small trees found in forest management inventory data (or ‘worst-case scenario’, i.e. c. 12.5%, 12.5% and 25% sampling for diameter classes 10–20 cm, 20–30 cm and 30–40 cm, respectively). While within-plot AGB variation induced by the sole small tree simulation function was high in this extreme sampling scenario (with an average plot CV of 7.7% (95% CI: 3.4–13.9), Fig. 6a), it led to a very slight estimation bias across plots (slope of the MA regression: 0.97 [0.95–0.99], Fig. 8).

### Validation of the biomass correction model for trees in diameter class 10–20 cm (specific function 3)

**Fig. 9:**
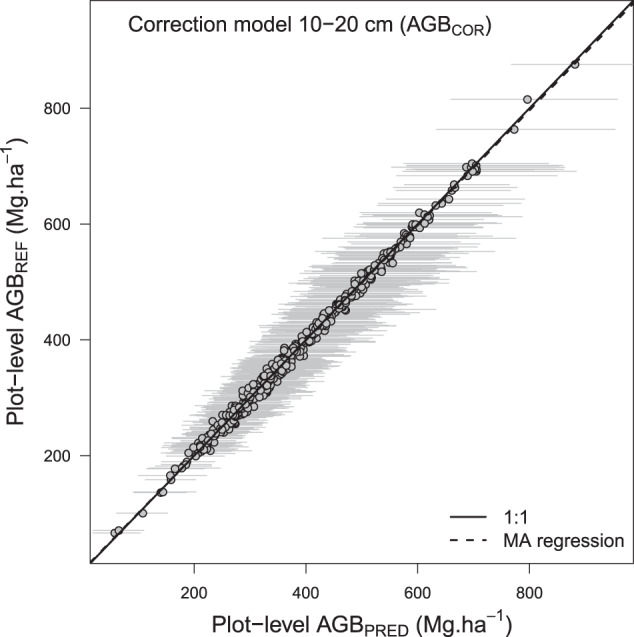
Results of the AGB correction model for trees in diameter class 10–20 cm. AGB_REF_ corresponds to an average of 1000 plots AGB simulations while propagating wood density, height-diameter and allometric model errors. In AGB_PRED_ (mean ± SD), the error associated with the AGB correction model for trees in diameter class 10–20 cm is also propagated. Grey dots and bars represent the mean and 95% confidence interval of plots biomass, respectively.

We removed trees in the 10-20 cm diameter class from scientific inventories and computed plot AGB using the AGB_10-20_ model for that class. The sole biomass correction model propagated an uncertainty of 6.4% on plot AGB estimates (95% CI: 3.3–11.5; Fig. 6a) and led to accurate AGB estimates across plots (slope of the MA regression: 0.99 [0.99–1.00], Fig. 9).

### Assessment of pixel-level uncertainty

**Fig. 10:**
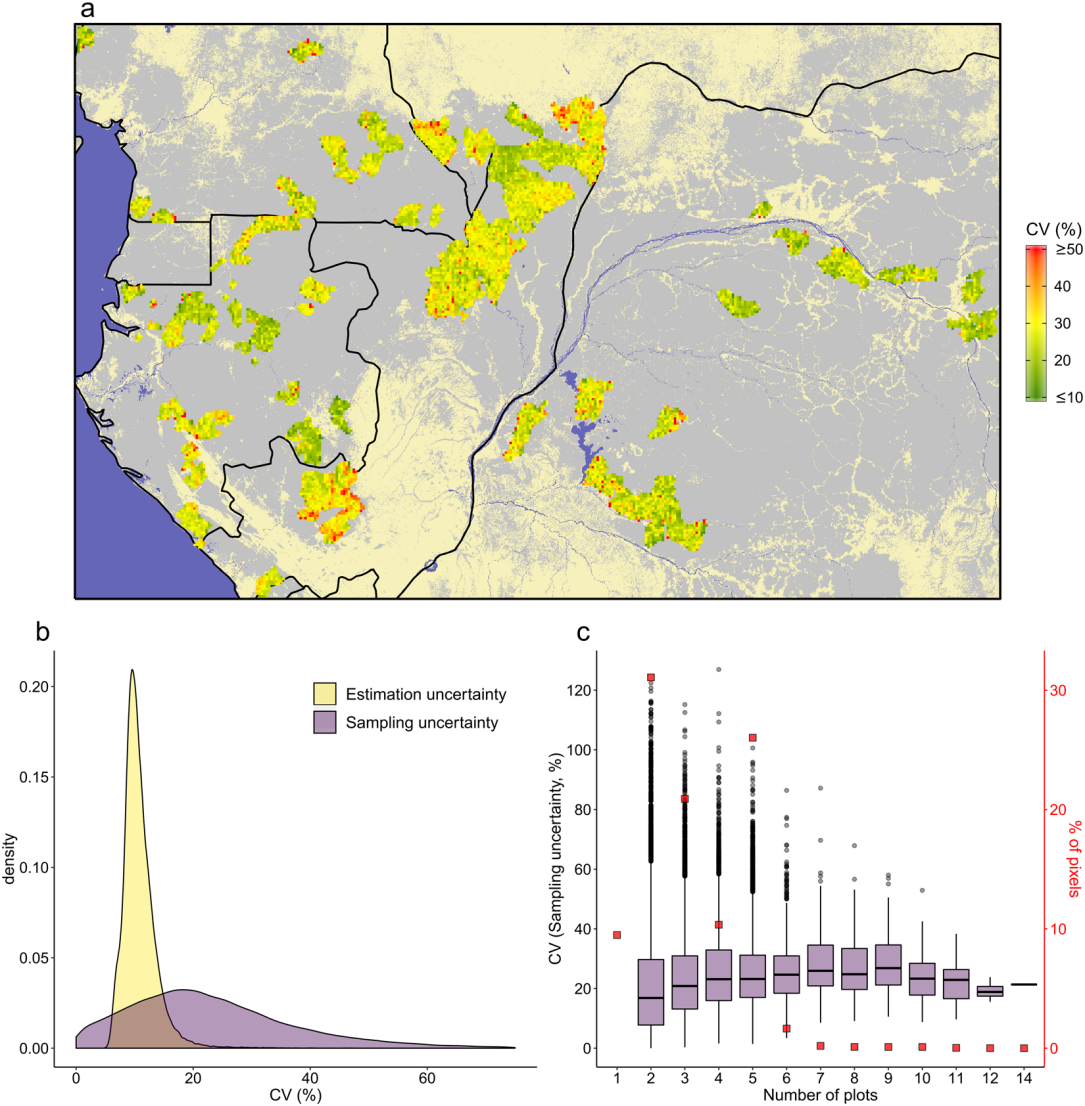
Uncertainties on pixel-level AGB estimates. (**a**) Total uncertainty on 1-km pixels AGB estimates (reported as a coefficient of variation, CV, in %), aggregated into 5-km pixels (for visual purpose), is depicted in a green-to-red color gradient and underlaid by the spatial distribution of moist forests^21^ (grey) and countries borders (black). (**b**) Frequency distribution of estimation and sampling uncertainties across pixels. (**c**) Distribution of sampling uncertainty by categories of pixels. Categories reflect the number of forest management plots available by pixel to compute pixel’s AGB. The proportion of each category in the CoFor dataset (in %) is represented with red square symbols.

Once aggregated into 1-km pixels, the total uncertainty (CV) on pixel AGB estimates was 26.3% on average and mostly comprised between 9.3% (quantile 2.5) and 61.1% (quantile 97.5). The spatial distribution of pixels total CV shows some clusters of pixels with lower uncertainty level (Fig. 10a), generally corresponding to high-biomass areas (as illustrated in Fig. 1). Pixel total CV was largely dominated by the sampling uncertainty (i.e. the inter-plot variance within pixels, Fig. 10b). When considered separately, the sampling and estimation (i.e. the mean intra-plot variance within pixels) uncertainties represented a mean CV (±sd) of 23.5% ± 14.9% and 11.8% ± 2.9%, respectively. While the sampling uncertainty did not depend strongly to the number of plots per pixel (Fig. 10c), the associated confidence interval on pixel mean AGB decreased with the squared root of the number of plots, from c. 26.7% to 20.7% and 14.9% for 3, 6 and 10 plots, respectively.

## Usage Notes

Given its size and regional coverage, this dataset should be particularly useful to the community of remote-sensing scientists working on tropical forest biomass. Developing, validating or comparing large-scale remote sensing models of tropical forest biomass is made difficult by the absence of standard, readily available large scale reference datasets^eg.^^36^. While the Global Ecosystem Dynamics Investigation (GEDI) sensor onboard the international space station will provide billions of discrete biomass estimations across the globe for the early 2020s^37^, the only reference data currently available for the first decade of the century are derived from the Geoscience Laser Altimeter System (GLAS). Biomass estimations computed from the CoFor dataset constitute the unique alternative to those from GLAS for large-scale remote-sensing applications in the central African region for this period. This dataset could also be used to re-calibrate existing global biomass maps, which has proven useful to increase the precision of mean biomass estimations over regions or forest types of interest using design-based inference^38^. In that, the potential of these data to improve the precision of carbon stocks or carbon emission factors used in national or jurisdictional REDD + projects in central Africa could be investigated. These data are also relevant to study the sensitivity of forest carbon to environmental conditions, which could provide insights into the potential response of forest carbon to climate change, a major source of uncertainty in current global climate models. By providing a decomposition of pixels’ forest biomass into its structural (number of trees, mean tree diameter) and compositional (i.e. mean wood density) characteristics at plot-level, we also expect these data to be useful to study local and regional variation patterns. Wood density for instance cannot be detected and mapped over large scales with current remote sensing data types. The data released here could thus be used, just like pixels biomass, as a calibration/validation set for wood density spatial models based on environmental data, or to derive ecosystem-levels means.

It should be noted that forest biomass in CoFor sample plots is based on stem diameter distribution and species averaged tree wood densities, while tree heights have been predicted from a regional allometry model. Tree diameter-height allometry varies locally^39^, and the regional model we used is known to produce biased estimates at some sites^40^. This error will thus propagate to plot biomass estimations. A similar pattern of opposite errors can be expected from GLAS-derived biomass estimations, which are based on height measurements with no information on the underlying stem diameters.

## Supplementary information

Supplementary Figure 1

## Data Availability

All analyses were performed in R (v.3.6.2). The BIOMASS R-package is an open source library available from the CRAN R repository. Codes associated to *specific functions* are available upon request.
