# Supplementary material for: A map of African humid tropical forest aboveground biomass derived from management inventories
Source: Sci Data. 2020 Jul 8;7:221. doi: 10.1038/s41597-020-0561-0 (PMC7343822; doi:10.1038/s41597-020-0561-0)
Supplement: Supplementary file 1 — Supplementary Figure 1 [file 41597_2020_561_MOESM1_ESM.pdf]

Supplementary Figures.

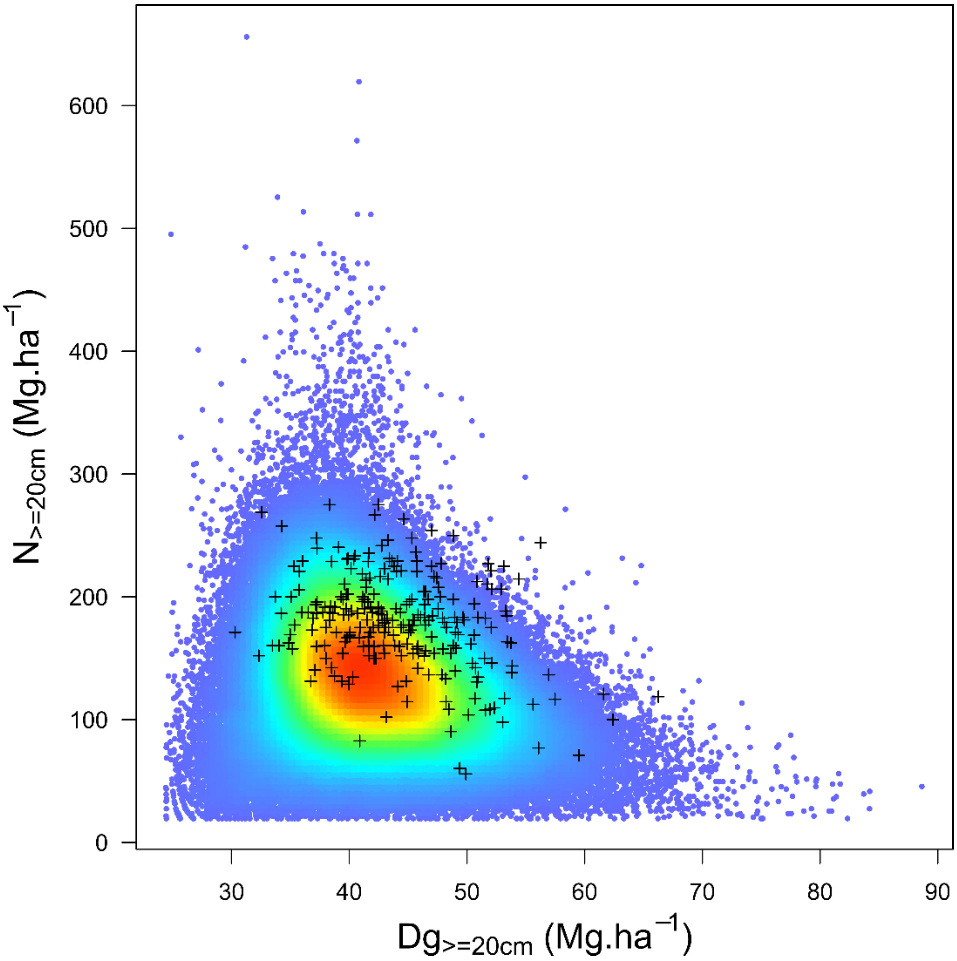

Supplementary Figure 1. Range of forest structural parameters in 0.5-ha plots from the CoFor (heat color gradient) and scientific (black crosses) datasets. $N_{>=20}$ and $Dg_{>=20}$ stands for the number of trees and the quadratic mean tree diameter for trees with diameter at breast height greater or equal than 20 cm, respectively.
